# Supplementary material for: Predicting no-shows at outpatient appointments in internal medicine using machine learning models
Source: PeerJ Comput Sci. 2025 Apr 22;11:e2762. doi: 10.7717/peerj-cs.2762 (PMC12190658; doi:10.7717/peerj-cs.2762)
Supplement: Supplemental Information 1 — Tables of statistical description of the data generated by the balancing methods [file peerj-cs-11-2762-s001.pdf]

Table 3. Patient characteristics with SMOTE balancing

Characteristic	Overall, N = 19,3941	Absences, N = 9,6971	Attendances, N = 9,6971	p-value2	q-value3
**Age**				<0.001	<0.001
Mean (SD)	49.34 (17.08)	47.37 (16.91)	51.31 (17.01)		
Median (IQR)	48.00 (36.00, 61.00)	45.00 (34.00, 58.00)	52.00 (38.00, 63.00)		
**Sex**				<0.001	<0.001
Female	13,046.00 (67.37%)	6,998.00 (72.38%)	6,048.00 (62.37%)		
Male	6,320.00 (32.63%)	2,671.00 (27.62%)	3,649.00 (37.63%)		
Unknown	28	28	0		
**Type of insurance coverage**					
0: ARL	537.00 (2.77%)	140.00 (1.44%)	397.00 (4.09%)		
1: Contributory	1,535.00 (7.91%)	421.00 (4.34%)	1,114.00 (11.49%)		
2: Subsidized	224.00 (1.15%)	200.00 (2.06%)	24.00 (0.25%)		
3: Prepaid	16,355.00 (84.33%)	8,576.00 (88.44%)	7,779.00 (80.22%)		
4: Private	584.00 (3.01%)	308.00 (3.18%)	276.00 (2.85%)		
5: International Private	22.00 (0.11%)	14.00 (0.14%)	8.00 (0.08%)		
6: Other	10.00 (0.05%)	7.00 (0.07%)	3.00 (0.03%)		
7: Other Institutional	122.00 (0.63%)	30.00 (0.31%)	92.00 (0.95%)		
8: Prepaid International	5.00 (0.03%)	1.00 (0.01%)	4.00 (0.04%)		
**Number of diseases**				<0.001	<0.001
Mean (SD)	1.31 (1.07)	1.07 (1.01)	1.54 (1.09)		
Median (IQR)	1.00 (1.00, 2.00)	1.00 (0.00, 1.00)	1.00 (1.00, 2.00)		
**Recent hospitalizations**				<0.001	<0.001
Mean (SD)	0.03 (0.19)	0.03 (0.20)	0.04 (0.19)		
Median (IQR)	0.00 (0.00, 0.00)	0.00 (0.00, 0.00)	0.00 (0.00, 0.00)		
**Number of medications**				<0.001	<0.001
Mean (SD)	0.45 (1.13)	0.24 (0.79)	0.66 (1.36)		
Median (IQR)	0.00 (0.00, 0.00)	0.00 (0.00, 0.00)	0.00 (0.00, 1.00)		
**Hour of appointment**					
6:00 to 8:00	10.00 (0.10%)	4.00 (0.08%)	6.00 (0.12%)		
8:00 to 10:00	2,195.00 (22.72%)	1,131.00 (23.71%)	1,064.00 (21.75%)		
10:00 to 12:00	2,549.00 (26.38%)	1,187.00 (24.88%)	1,362.00 (27.84%)		
12:00 to 14:00	837.00 (8.66%)	421.00 (8.83%)	416.00 (8.50%)		
14:00 to 16:00	2,391.00 (24.75%)	1,236.00 (25.91%)	1,155.00 (23.61%)		
16:00 to 18:00	1,680.00 (17.39%)	791.00 (16.58%)	889.00 (18.17%)		
**Day of the appointment**				<0.001	<0.001
Sunday	3,571.00 (18.52%)	1,943.00 (20.28%)	1,628.00 (16.79%)		
Monday	3,375.00 (17.51%)	2,087.00 (21.78%)	1,288.00 (13.28%)		
Tuesday	3,045.00 (15.79%)	1,825.00 (19.05%)	1,220.00 (12.58%)		
Wednesday	3,017.00 (15.65%)	1,696.00 (17.70%)	1,321.00 (13.62%)		
Thursday	2,302.00 (11.94%)	1,094.00 (11.42%)	1,208.00 (12.46%)		
Friday	1,877.00 (9.74%)	528.00 (5.51%)	1,349.00 (13.91%)		
Saturday	2,092.00 (10.85%)	409.00 (4.27%)	1,683.00 (17.36%)		
**Month of the appointment**				<0.001	<0.001
January	1,263.00 (6.53%)	560.00 (5.80%)	703.00 (7.25%)		
February	1,216.00 (6.29%)	627.00 (6.50%)	589.00 (6.07%)		
March	1,507.00 (7.79%)	760.00 (7.88%)	747.00 (7.70%)		
April	1,580.00 (8.17%)	914.00 (9.47%)	666.00 (6.87%)		
May	1,808.00 (9.35%)	879.00 (9.11%)	929.00 (9.58%)		
June	2,047.00 (10.58%)	949.00 (9.84%)	1,098.00 (11.32%)		
July	2,257.00 (11.67%)	931.00 (9.65%)	1,326.00 (13.67%)		
August	3,129.00 (16.17%)	1,347.00 (13.96%)	1,782.00 (18.38%)		
September	2,972.00 (15.36%)	1,115.00 (11.56%)	1,857.00 (19.15%)		
October	570.00 (2.95%)	570.00 (5.91%)	0.00 (0.00%)		
November	606.00 (3.13%)	606.00 (6.28%)	0.00 (0.00%)		
December	390.00 (2.02%)	390.00 (4.04%)	0.00 (0.00%)		
**Interval creation to assignment**				<0.001	<0.001
Mean (SD)	25.54 (28.39)	25.61 (25.35)	25.47 (31.14)		
Median (IQR)	20.00 (8.00, 35.00)	20.00 (8.00, 34.00)	19.00 (7.00, 35.00)		
**Number of previous attendances**				<0.001	<0.001
Mean (SD)	2.56 (2.23)	2.46 (2.34)	2.67 (2.11)		
Median (IQR)	2.00 (1.00, 3.00)	2.00 (1.00, 3.00)	2.00 (1.00, 4.00)		
**Number of previous absences**				<0.001	<0.001
Mean (SD)	2.14 (3.42)	2.72 (3.64)	1.56 (3.08)		
Median (IQR)	1.00 (0.00, 3.00)	2.00 (1.00, 3.00)	1.00 (0.00, 2.00)		

Table 2. Patient characteristics with RandomSplit balancing

Characteristic	Overall, N = 12,868[1]	Absences, N = 6,434[1]	Attendances, N = 6,434[1]	p-value[2]	q-value3
**Age**				<0.001	<0.001
Mean (SD)	49.61 (17.11)	47.70 (17.03)	51.51 (16.99)		
Median (IQR)	49.00 (36.00, 61.00)	46.00 (35.00, 59.00)	52.00 (39.00, 63.00)		
**Sex**				0.070	0.088
Female	8,000.00 (62.33%)	3,940.00 (61.55%)	4,060.00 (63.10%)		
Male	4,835.00 (37.67%)	2,461.00 (38.45%)	2,374.00 (36.90%)		
Unknown	33	33	0		
**Type of insurance coverage**					
0: ARL	355.00 (2.76%)	101.00 (1.57%)	254.00 (3.95%)		
1: Contributory	1,022.00 (7.94%)	298.00 (4.63%)	724.00 (11.25%)		
2: Subsidized	32.00 (0.25%)	11.00 (0.17%)	21.00 (0.33%)		
3: Prepaid	10,855.00 (84.36%)	5,659.00 (87.95%)	5,196.00 (80.76%)		
4: Private	496.00 (3.85%)	321.00 (4.99%)	175.00 (2.72%)		
5: International Private	8.00 (0.06%)	4.00 (0.06%)	4.00 (0.06%)		
6: Other	4.00 (0.03%)	4.00 (0.06%)	0.00 (0.00%)		
7: Other Institutional	94.00 (0.73%)	35.00 (0.54%)	59.00 (0.92%)		
8: Prepaid International	2.00 (0.02%)	1.00 (0.02%)	1.00 (0.02%)		
**Number of diseases**				<0.001	<0.001
Mean (SD)	1.39 (1.12)	1.23 (1.11)	1.56 (1.11)		
Median (IQR)	1.00 (1.00, 2.00)	1.00 (1.00, 2.00)	1.00 (1.00, 2.00)		
**Recent hospitalizations**				0.3	0.3
Mean (SD)	0.04 (0.22)	0.04 (0.24)	0.04 (0.19)		
Median (IQR)	0.00 (0.00, 0.00)	0.00 (0.00, 0.00)	0.00 (0.00, 0.00)		
**Number of medications**				<0.001	<0.001
Mean (SD)	0.50 (1.19)	0.32 (0.93)	0.68 (1.38)		
Median (IQR)	0.00 (0.00, 0.00)	0.00 (0.00, 0.00)	0.00 (0.00, 1.00)		
**Hour of appointment**					
6:00 to 8:00	8.00 (0.12%)	3.00 (0.10%)	5.00 (0.15%)		
8:00 to 10:00	1,466.00 (22.86%)	720.00 (22.86%)	746.00 (22.86%)		
10:00 to 12:00	1,636.00 (25.51%)	749.00 (23.79%)	887.00 (27.18%)		
12:00 to 14:00	463.00 (7.22%)	181.00 (5.75%)	282.00 (8.64%)		
14:00 to 16:00	1,580.00 (24.64%)	824.00 (26.17%)	756.00 (23.17%)		
16:00 to 18:00	1,259.00 (19.64%)	672.00 (21.34%)	587.00 (17.99%)		
**Day of the appointment**				<0.001	<0.001
Sunday	2,363.00 (18.55%)	1,224.00 (19.41%)	1,139.00 (17.70%)		
Monday	2,096.00 (16.45%)	1,246.00 (19.76%)	850.00 (13.21%)		
Tuesday	1,904.00 (14.95%)	1,129.00 (17.91%)	775.00 (12.05%)		
Wednesday	2,047.00 (16.07%)	1,148.00 (18.21%)	899.00 (13.97%)		
Thursday	1,622.00 (12.73%)	841.00 (13.34%)	781.00 (12.14%)		
Friday	1,206.00 (9.47%)	348.00 (5.52%)	858.00 (13.34%)		
Saturday	1,501.00 (11.78%)	369.00 (5.85%)	1,132.00 (17.59%)		
**Month of the appointment**				<0.001	<0.001
January	800.00 (6.23%)	360.00 (5.63%)	440.00 (6.84%)		
February	801.00 (6.24%)	401.00 (6.27%)	400.00 (6.22%)		
March	985.00 (7.68%)	488.00 (7.63%)	497.00 (7.72%)		
April	1,037.00 (8.08%)	591.00 (9.24%)	446.00 (6.93%)		
May	1,189.00 (9.27%)	574.00 (8.97%)	615.00 (9.56%)		
June	1,295.00 (10.09%)	590.00 (9.22%)	705.00 (10.96%)		
July	1,444.00 (11.25%)	553.00 (8.64%)	891.00 (13.85%)		
August	2,014.00 (15.70%)	838.00 (13.10%)	1,176.00 (18.28%)		
September	2,091.00 (16.30%)	827.00 (12.93%)	1,264.00 (19.65%)		
October	378.00 (2.95%)	378.00 (5.91%)	0.00 (0.00%)		
November	407.00 (3.17%)	407.00 (6.36%)	0.00 (0.00%)		
December	391.00 (3.05%)	391.00 (6.11%)	0.00 (0.00%)		
**Interval creation to assignment**				0.088	0.10
Mean (SD)	25.63 (25.88)	25.59 (25.35)	25.68 (26.40)		
Median (IQR)	20.00 (8.00, 35.00)	20.00 (8.00, 34.00)	19.00 (7.00, 35.00)		
**Number of previous attendances**				<0.001	<0.001
Mean (SD)	2.68 (2.33)	2.69 (2.50)	2.68 (2.16)		
Median (IQR)	2.00 (1.00, 4.00)	2.00 (1.00, 4.00)	2.00 (1.00, 4.00)		
**Number of previous absences**				<0.001	<0.001
Mean (SD)	2.27 (3.58)	2.99 (3.96)	1.54 (2.99)		
Median (IQR)	1.00 (0.00, 3.00)	2.00 (1.00, 4.00)	1.00 (0.00, 2.00)		

Table 1. Patient characteristics with ADASYN balancing

Characteristic	Overall, N = 19,515[1]	Absences, N = 9,818[1]	Attendances, N = 9,697[1]	p-value[2]	q-value[3]
**Age**				<0.001	<0.001
Mean (SD)	49.80 (17.07)	48.32 (17.00)	51.31 (17.01)		
Median (IQR)	49.00 (37.00, 61.00)	47.00 (35.00, 60.00)	52.00 (38.00, 63.00)		
**Sex**				<0.001	<0.001
Female	13,061.00 (67.03%)	7,013.00 (71.65%)	6,048.00 (62.37%)		
Male	6,424.00 (32.97%)	2,775.00 (28.35%)	3,649.00 (37.63%)		
Unknown	30	30	0		
**Type of insurance coverage**					
0: ARL	538.00 (2.76%)	141.00 (1.44%)	397.00 (4.09%)		
1: Contributory	1,587.00 (8.13%)	473.00 (4.82%)	1,114.00 (11.49%)		
2: Subsidized	240.00 (1.23%)	216.00 (2.20%)	24.00 (0.25%)		
3: Prepaid	16,407.00 (84.07%)	8,628.00 (87.88%)	7,779.00 (80.22%)		
4: Private	591.00 (3.03%)	315.00 (3.21%)	276.00 (2.85%)		
5: International Private	18.00 (0.09%)	10.00 (0.10%)	8.00 (0.08%)		
6: Other	7.00 (0.04%)	4.00 (0.04%)	3.00 (0.03%)		
7: Other Institutional	122.00 (0.63%)	30.00 (0.31%)	92.00 (0.95%)		
8: Prepaid International	5.00 (0.03%)	1.00 (0.01%)	4.00 (0.04%)		
**Number of diseases**				<0.001	<0.001
Mean (SD)	1.30 (1.07)	1.07 (1.00)	1.54 (1.09)		
Median (IQR)	1.00 (1.00, 2.00)	1.00 (0.00, 1.00)	1.00 (1.00, 2.00)		
**Recent hospitalizations**				<0.001	<0.001
Mean (SD)	0.03 (0.19)	0.03 (0.20)	0.04 (0.19)		
Median (IQR)	0.00 (0.00, 0.00)	0.00 (0.00, 0.00)	0.00 (0.00, 0.00)		
**Number of medications**				<0.001	<0.001
Mean (SD)	0.45 (1.13)	0.24 (0.80)	0.66 (1.36)		
Median (IQR)	0.00 (0.00, 0.00)	0.00 (0.00, 0.00)	0.00 (0.00, 1.00)		
**Hour of appointment**				<0.001	<0.001
6:00 to 8:00	11.00 (0.11%)	5.00 (0.10%)	6.00 (0.12%)		
8:00 to 10:00	2,258.00 (23.30%)	1,194.00 (24.89%)	1,064.00 (21.75%)		
10:00 to 12:00	2,549.00 (26.31%)	1,187.00 (24.74%)	1,362.00 (27.84%)		
12:00 to 14:00	877.00 (9.05%)	461.00 (9.61%)	416.00 (8.50%)		
14:00 to 16:00	2,391.00 (24.68%)	1,236.00 (25.77%)	1,155.00 (23.61%)		
16:00 to 18:00	1,603.00 (16.54%)	714.00 (14.88%)	889.00 (18.17%)		
**Day of the appointment**				<0.001	<0.001
Sunday	3,561.00 (18.37%)	1,933.00 (19.94%)	1,628.00 (16.79%)		
Monday	3,336.00 (17.20%)	2,048.00 (21.13%)	1,288.00 (13.28%)		
Tuesday	3,050.00 (15.73%)	1,830.00 (18.88%)	1,220.00 (12.58%)		
Wednesday	3,060.00 (15.78%)	1,739.00 (17.94%)	1,321.00 (13.62%)		
Thursday	2,378.00 (12.26%)	1,170.00 (12.07%)	1,208.00 (12.46%)		
Friday	1,888.00 (9.74%)	539.00 (5.56%)	1,349.00 (13.91%)		
Saturday	2,117.00 (10.92%)	434.00 (4.48%)	1,683.00 (17.36%)		
**Month of the appointment**				<0.001	<0.001
January	1,297.00 (6.66%)	594.00 (6.08%)	703.00 (7.25%)		
February	1,275.00 (6.55%)	686.00 (7.02%)	589.00 (6.07%)		
March	1,527.00 (7.84%)	780.00 (7.98%)	747.00 (7.70%)		
April	1,647.00 (8.46%)	981.00 (10.04%)	666.00 (6.87%)		
May	1,844.00 (9.47%)	915.00 (9.37%)	929.00 (9.58%)		
June	2,079.00 (10.68%)	981.00 (10.04%)	1,098.00 (11.32%)		
July	2,297.00 (11.80%)	971.00 (9.94%)	1,326.00 (13.67%)		
August	3,143.00 (16.15%)	1,361.00 (13.93%)	1,782.00 (18.38%)		
September	2,985.00 (15.33%)	1,128.00 (11.55%)	1,857.00 (19.15%)		
October	533.00 (2.74%)	533.00 (5.46%)	0.00 (0.00%)		
November	499.00 (2.56%)	499.00 (5.11%)	0.00 (0.00%)		
December	341.00 (1.75%)	341.00 (3.49%)	0.00 (0.00%)		
**Interval creation to assignment**				0.002	0.002
Mean (SD)	25.64 (28.69)	25.81 (26.05)	25.47 (31.14)		
Median (IQR)	19.00 (7.00, 35.00)	20.00 (8.00, 34.00)	19.00 (7.00, 35.00)		
**Number of previous attendances**				<0.001	<0.001
Mean (SD)	2.55 (2.22)	2.44 (2.33)	2.67 (2.11)		
Median (IQR)	2.00 (1.00, 3.00)	2.00 (1.00, 3.00)	2.00 (1.00, 4.00)		
**Number of previous absences**				<0.001	<0.001
Mean (SD)	2.09 (3.39)	2.61 (3.60)	1.56 (3.08)		
Median (IQR)	1.00 (0.00, 3.00)	1.00 (1.00, 3.00)	1.00 (0.00, 2.00)		
