# Supplementary material for: Predicting no-shows at outpatient appointments in internal medicine using machine learning models
Source: PeerJ Comput Sci. 2025 Apr 22;11:e2762. doi: 10.7717/peerj-cs.2762 (PMC12190658; doi:10.7717/peerj-cs.2762)
Supplement: Supplemental Information 2 — Figures of the shap values for the AdaBoost, Bagging and GradientBoosting models for each data preprocessing. [file peerj-cs-11-2762-s002.pdf]

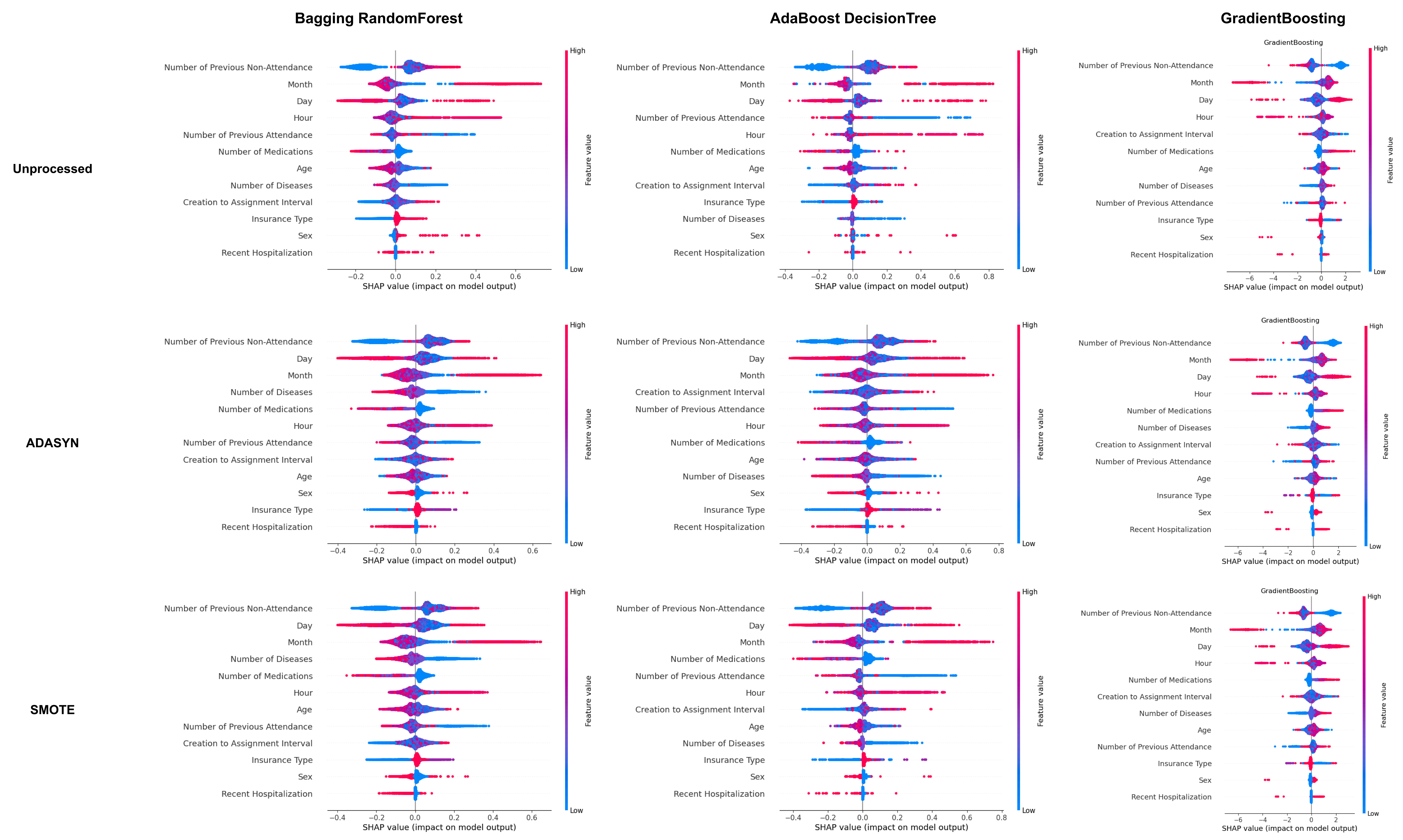

Bagging RandomForest
AdaBoost DecisionTree
GradientBoosting
Unprocessed
ADASYN
SMOTE
GradientBoosting
Number of Previous Non-Attendance
Month
Day
Hour
Number of Previous Attendance
Number of Medications
Age
Number of Diseases
Creation to Assignment Interval
Insurance Type
Sex
Recent Hospitalization
SHAP value (impact on model output)
Feature value
High
Low
