# Supplementary material for: Predicting no-shows at outpatient appointments in internal medicine using machine learning models
Source: PeerJ Comput Sci. 2025 Apr 22;11:e2762. doi: 10.7717/peerj-cs.2762 (PMC12190658; doi:10.7717/peerj-cs.2762)

Unprocessed

StandardScaler

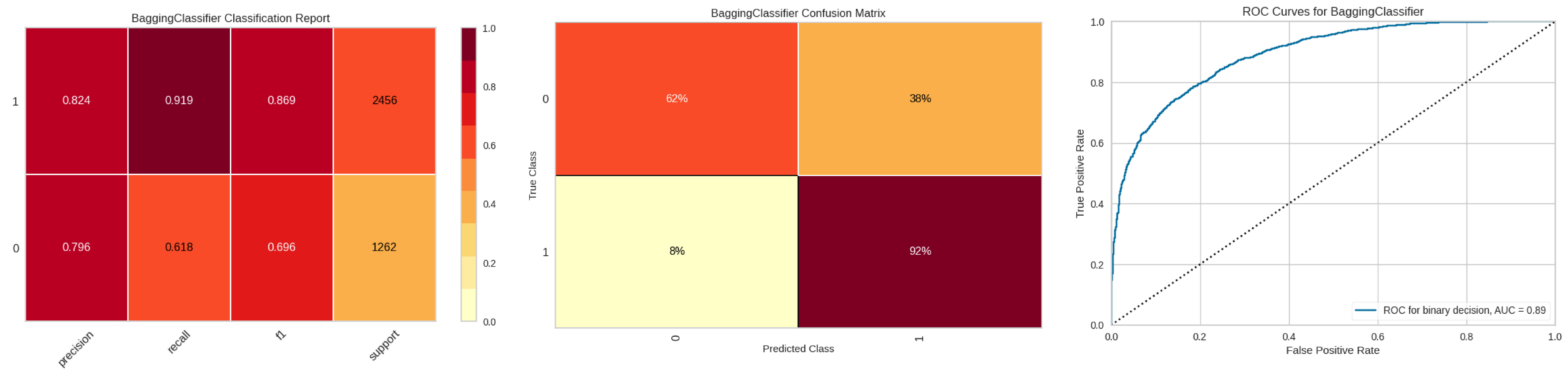

Non StandardScaler

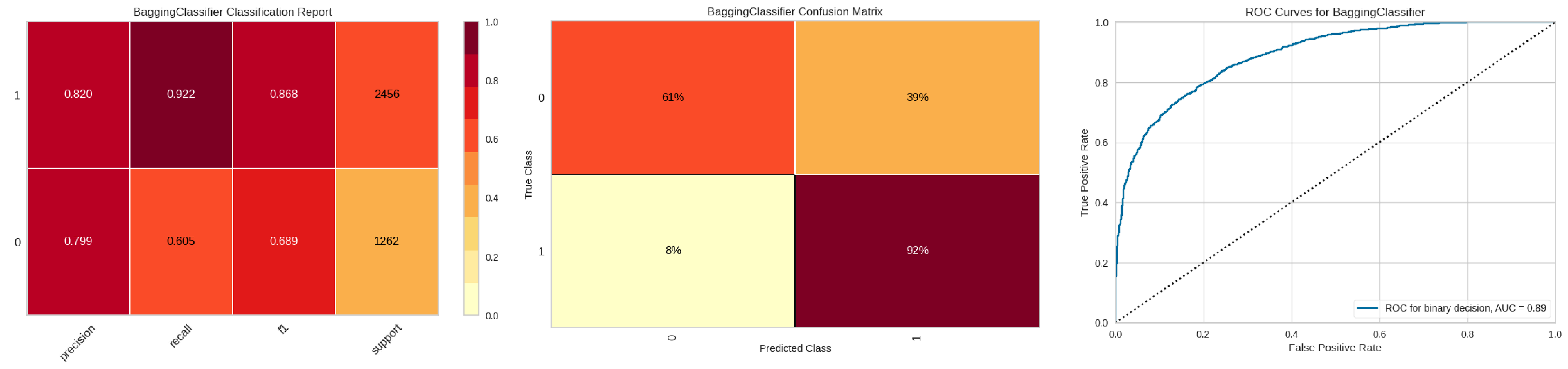

RandomSplit

StandardScaler

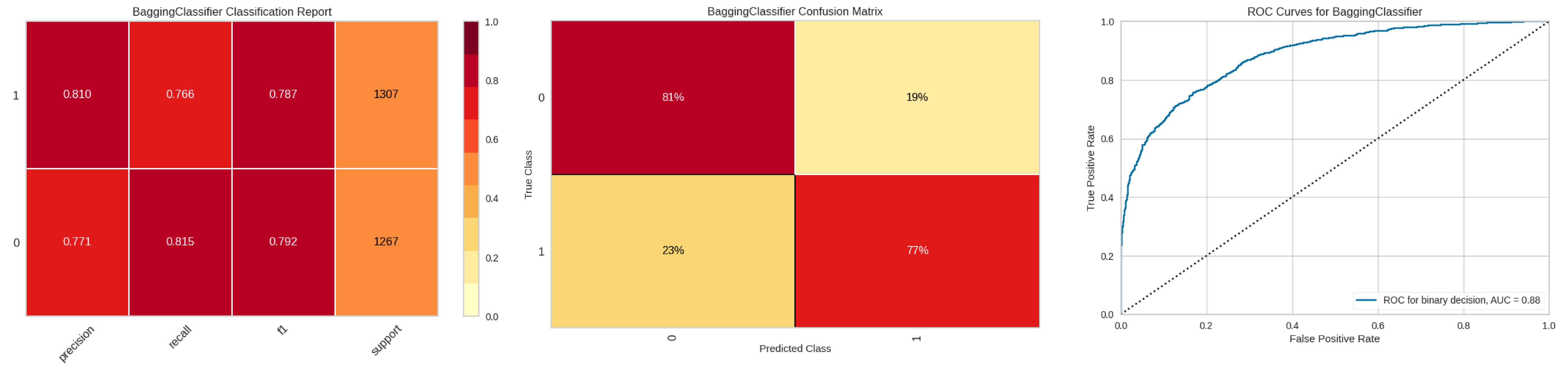

Non StandardScaler

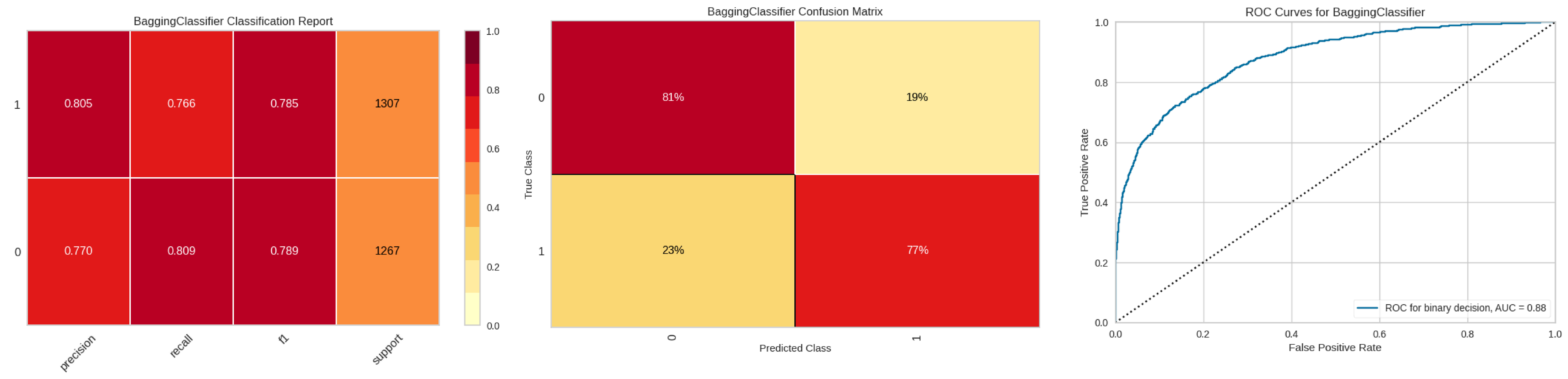

ADASYN

StandardScaler

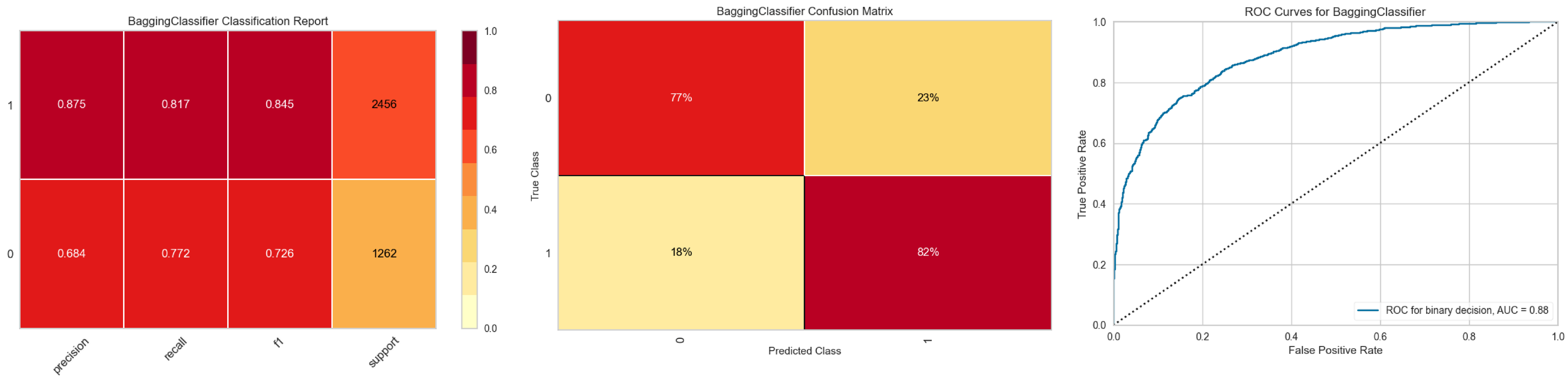

Non StandardScaler

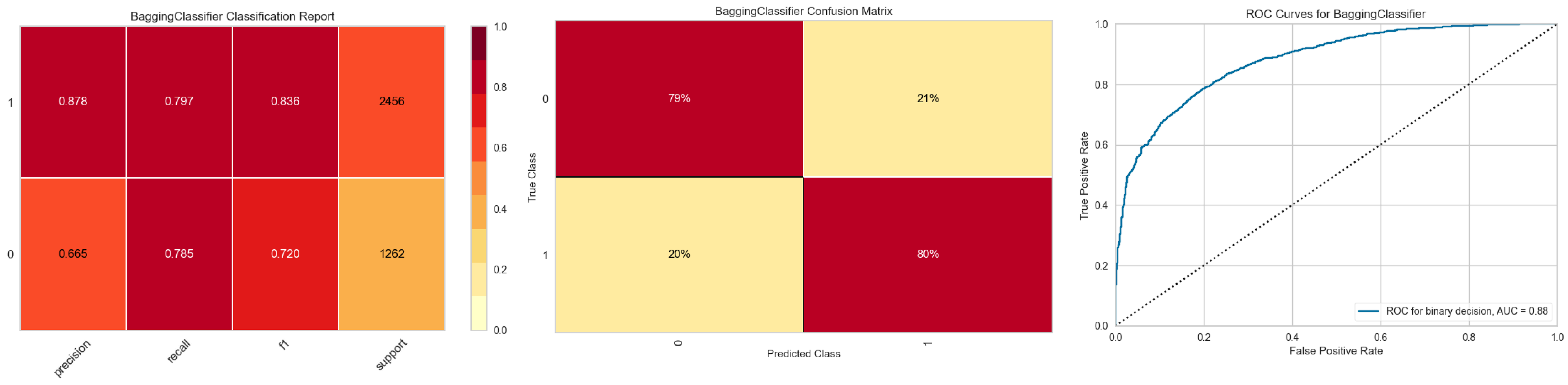

SMOTE

StandardScaler

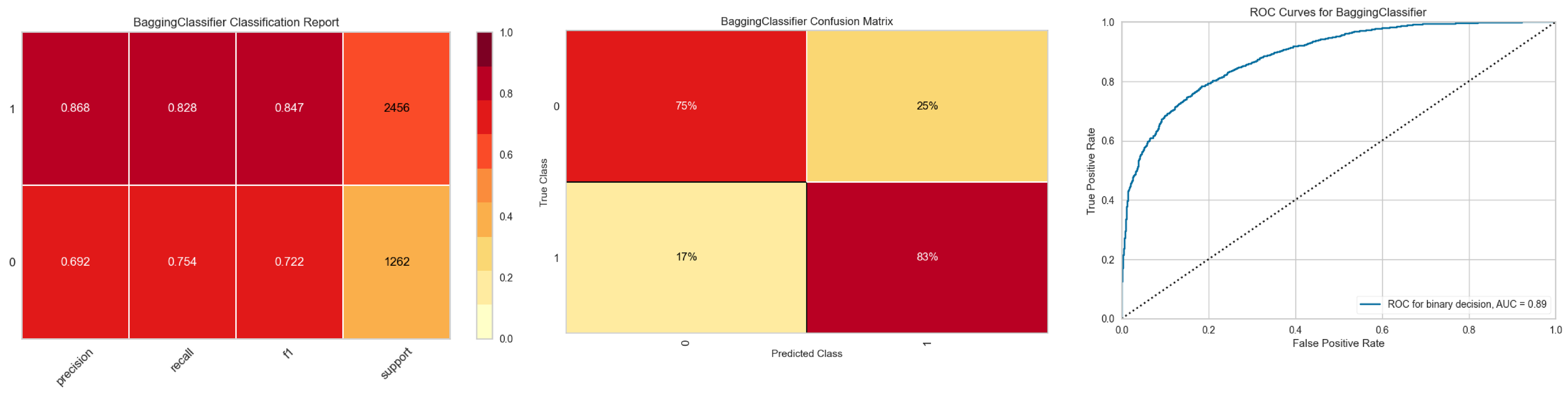

Non StandardScaler

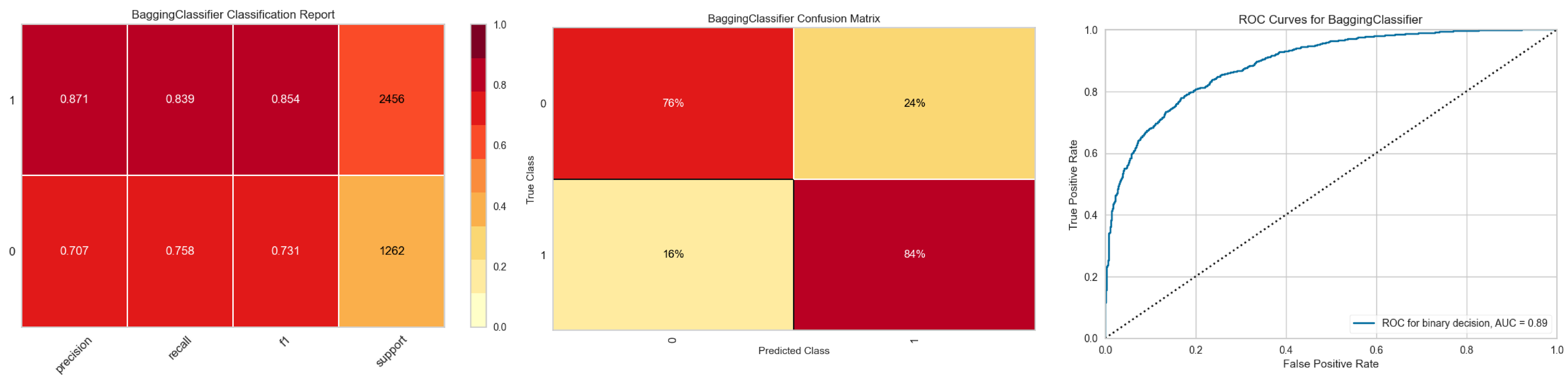

Supplement: Supplemental Information 3 — Bagging RandomForest model metrics per data preprocessing [file peerj-cs-11-2762-s003.pdf]
